# Course of lactate, pH and base excess for prediction of mortality in medical intensive care patients

**DOI:** 10.1371/journal.pone.0261564

**Published:** 2021-12-20

**Authors:** Anja Schork, Kathrin Moll, Michael Haap, Reimer Riessen, Robert Wagner

**Affiliations:** 1 Division of Endocrinology, Diabetology, and Nephrology, Department of Internal Medicine IV, University Hospital Tübingen, Tübingen, Germany; 2 Institute of Diabetes Research and Metabolic Diseases (IDM) of the Helmholtz Center Munich at the University of Tübingen, Tübingen, Germany; 3 German Center for Diabetes Research (DZD e.V.), Neuherberg, Germany; 4 Department of Internal Medicine, Medical Intensive Care Unit, University Hospital Tübingen, Tübingen, Germany; Heidelberg University Hospital, GERMANY

## Abstract

**Introduction:**

As base excess had shown superiority over lactate as a prognostic parameter in intensive care unit (ICU) surgical patients we aimed to evaluate course of lactate, base excess and pH for prediction of mortality of medical ICU patients.

**Materials and methods:**

For lactate, pH and base excess, values at the admission to ICU, at 24 ± 4 hours, maximum or minimum in the first 24 hours and in 24–48 hours after admission were collected from all patients admitted to the Medical ICU of the University Hospital Tübingen between January 2016 until December 2018 (N = 4067 at admission, N = 1715 with ICU treatment > 48 h) and investigated for prediction of in-hospital-mortality.

**Results:**

Mortality was 22% and significantly correlated with all evaluated parameters. Strongest predictors of mortality determined by ROC were maximum lactate in 24 h (AUROC 0.74, cut off 2.7 mmol/L, hazard ratio of risk group with value > cut off 3.20) and minimum pH in 24 h (AUROC 0.71, cut off 7.31, hazard ratio for risk group 2.94). Kaplan Meier Curves stratified across these cut offs showed early and clear separation. Hazard ratios per standard deviation increase were highest for maximum lactate in 24 h (HR 1.65), minimum base excess in 24 h (HR 1.56) and minimum pH in 24 h (HR 0.75).

**Conclusion:**

Lactate, pH and base excess were all suitable predictors of mortality in internal ICU patients, with maximum / minimum values in 24 and 24–48 h after admission altogether stronger predictors than values at admission. Base excess and pH were not superior to lactate for prediction of mortality.

## Introduction

Estimation of the mortality of patients at intensive care unit (ICU) is necessary for treatment planning and treatment decisions as well as for support and advice for the patient’s relatives. Various surrogate parameters and their significance for the assessment of mortality risk have been evaluated, in particular lactate as read-out of anaerobic metabolism and tissue perfusion [1, 2]. Elevated lactate level is common in patients admitted to ICU and a strong predictor of mortality in unselected ICU patients [3, 4] and lactate clearance was recently discovered as an even stronger parameter than initial lactate level for assessing mortality risk of critically ill patients [5, 6].

To account for the ability to buffer a metabolic (lactate) acidosis, parameters of acid-base balance, such as base excess or pH, could represent the body’s conditions as more general parameters than lactate. Acid-base parameters have recently been evaluated as parameters for estimation of mortality in different subgroups of patients: In patients after cardiac surgery, base excess at ICU admission was a stronger parameter for prediction of ICU mortality than lactate-levels [7]. Lactate, anion gap and base excess were interchangeable biomarkers of traumatic shock [8] and base excess was a strong predictor of mortality in a large cohort of trauma patients [9]. Bicarbonate and anion gap were associated with higher mortality in sepsis patients even if lactate levels were low [10]. Metabolic acidosis at admission to ICU and early pH changes correlated with higher mortality in a small Indian cohort of critically ill patients [11]. However, for evaluation of acid base parameters as predictors of mortality of patients requiring treatment at a medical ICU, there is still a lack of data. We therefore aimed to evaluate parameters of acid-base balance obtainable by blood gas analysis as predictors of mortality in critically ill medical patients.

## Materials and methods

### Patients and blood gas analysis

**Fig 1:**
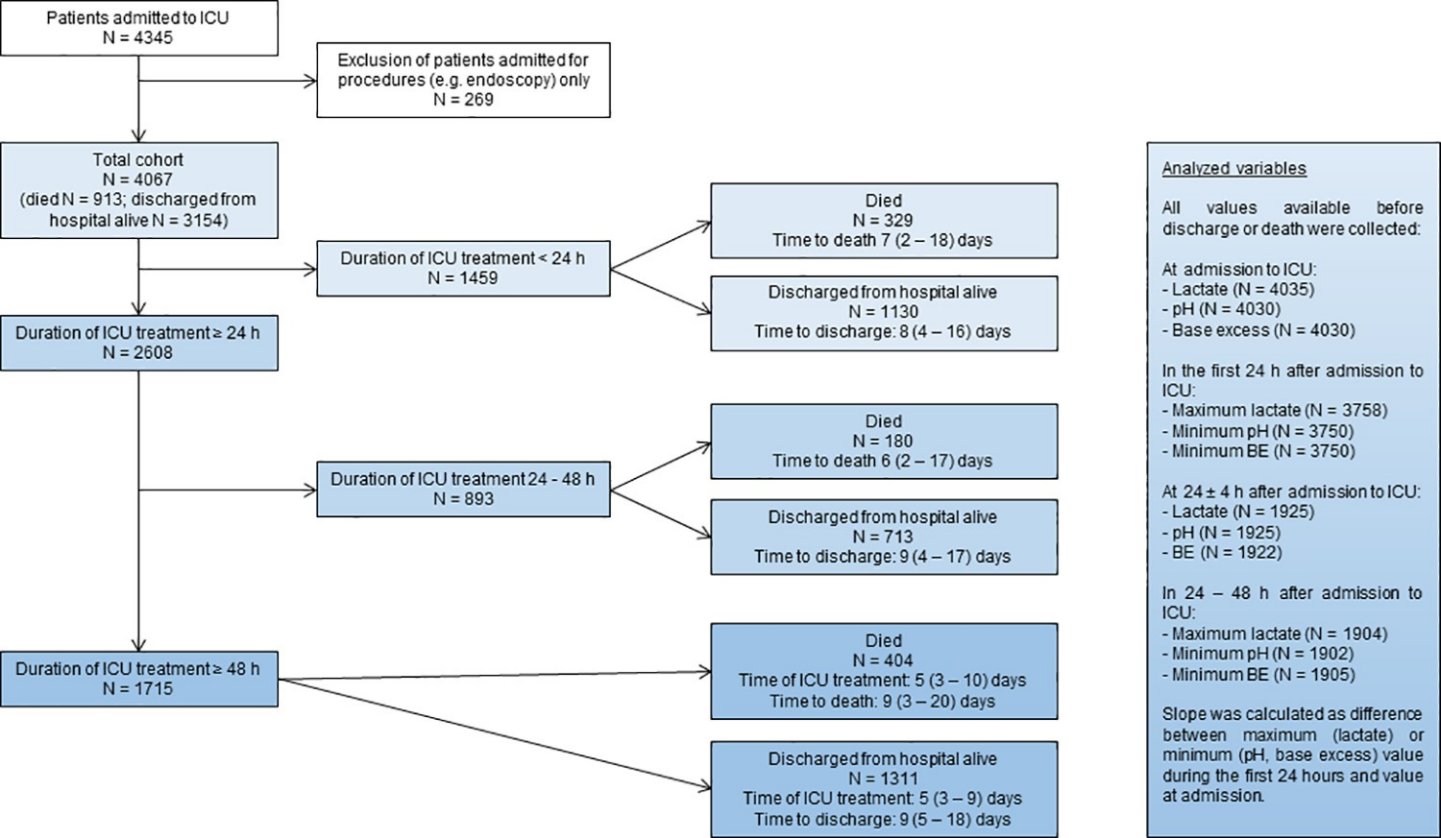
Flow chart study cohort and evaluated parameters. Fig 1 shows a flow chart on the outcome of patients with different duration of ICU treatment with an overview of available numbers of examined parameters at the respective time points.

The study was approved by the local ethics committee of the University of Tuebingen and the need for consent was waived for this retrospective analysis (139/2019B02). Data from all patients admitted to the Medical ICU of the University Hospital Tübingen between January 2016 until December 2018 was collected from the patient data management system (ICCA, Philips GmbH) of the University Hospital Tübingen and evaluated retrospectively. Age, gender and SAPS II score at admission to ICU and need for invasive ventilation or dialysis during the treatment at ICU were documented (we did not use SAPS III score due to too many missing values). Laboratory data obtained included base excess, pH and lactate from arterial or venous blood gas analysis at admission to ICU and during the first 48 hours after admission to ICU. All blood gas analyses were performed with a Radiometer ABL90 FLEX. The following parameters were evaluated as predictors of mortality (Fig 1): value at admission, value after 24 ± 4 hours, maximum (lactate) or minimum (pH, base excess) value in the first 24 hours and between 24–48 hours after admission and slope of lactate, pH and base excess. Slope of the variables was calculated as difference between maximum (lactate) or minimum (pH, base excess) value during the first 24 hours and value at admission. All analyses were performed with all available patient data at the respective time points.

Mortality was defined by the outcome at discharge from hospital to one of two categories: patients who died in hospital and patients who were discharged from hospital alive. Primary diagnosis and cause of death were classified into groups according to the recorded ICD-10 (International Statistical Classification of Diseases and Related Health Problems) classification.

### Statistical analysis

Statistical analyses were performed using R version 3.6.1, SAS JMP Pro 14.2.0 and MedCalc 19.1. Distributions are reported as number (n) and percent for categorical parameters. Median and interquartile range (IQ) are provided for continuous parameters. χ^2^ test (nominal variables) and Mann Whitney U test (continuous variables) were performed to test for differences between groups.

Computation of receiver operating characteristics (ROC, C-statistics) was performed to evaluate the ability of parameters to predict mortality, with determination of the cut off value by Youden index (J = sensitivity + (specificity– 1)), and the area under the receiver operating characteristics curve (AUROC) is reported. Hazard ratios were determined from Cox regression for risk groups divided by cut offs from ROC, or per increase of the variable of 1 standard deviation (SD). Kaplan Meier curves were constructed for groups stratified by cut-offs from C-statistics using log-rank test to test for differences. Statistical significance was determined by two-sided tests with an alpha of 0.05 (p < 0.05).

## Results

### Study cohort

A total number of 4067 patients was admitted to intensive care treatment at the medical ICU of the University Hospital Tübingen between January 2016 until December 2018 and included in the analysis as shown in Fig 1. N = 913 patients (22%) died after a median of 8 (interquartile range 2–18) days.

Causes of death in the cohort classified by ICD-10 category were I (‘diseases of circulatory system’, including stroke, intracranial bleeding, pulmonary embolism, myocardial infarction, cardiomyopathy, valvular diseases, cardiac arrhythmias; 21%), J (‘diseases of the respiratory system’, including pneumonia, chronic obstructive pulmonary disease; 16%), A + B (‘certain infectious and parasitic disease’, including sepsis; 16%), R57.0 (‘cardiogenic shock’; 13%), C + D (‘neoplasms and diseases of the blood and hematopoietic organs and certain disorders involving the immune system’; 11%), K (‘diseases of the digestive system’, including alcoholic cirrhosis of the liver; 7%), and R57.2 (‘septic shock’; 6%); in 10% of patients, cause of death could not be classified into one of these categories.

The full characteristics of the total study cohort and of the patients who died or were discharged from hospital alive are listed in Table 1. Patients who died or were discharged from hospital alive were not different regarding age and gender, but showed significant differences in SAPS II Score, need for invasive ventilation or dialysis, and base excess, lactate and pH values (Table 1). Patients could be assigned into groups based on primary diagnoses as follows (Table 1): Infectious = ICD R57.2 + A + B (n = 290), cardiac = ICD R57.0 + I (n = 1482), respiratory = ICD J (n = 726), malignant = ICD C + D (n = 421) and other / uncertain (n = 1148). In our ICU cohort, there was a relevant number of patients with a short duration of ICU treatment, N = 1715 patients (42% of the total cohort) required ICU treatment > 48 h. Table 2 compares characteristics of patients with duration of ICU treatment >48 h compared to patients with short term ICU treatment <48 h. Patients who required ICU treatment > 48 h had higher SAPS II score, and more often required invasive ventilation or dialysis. There was no significant difference in mortality, age, gender, primary diagnosis group, and base excess, lactate and pH at ICU admission between patients with ICU treatment duration > 48 h or < 48 h.

**Table 1:** Characteristics of study cohort.

	Total cohort	Patients who died	Patients discharged from hospital alive	p value
Age	68 (55–78)	71 (60–80)	67 (53–77)	<0.0001
Gender (m, male; f, female)	m 2270 (56%)	m 511 (56%)	m 1757 (56%)	0.9151
	f 1797 (44%)	f 402 (44%)	f 1395 (44%)	
SAPS II score, points	42 (28–54)	44 (29–57)	41 (28–53)	0.0383
SAPS II estimated mortality rate, %	29 (9–55)	32 (10–62)	27 (9–53)	0.0377
Invasive ventilation	1894 (47%)	480 (53%)	1416 (45%)	<0.0001
Dialysis	531 (13%)	143 (16%)	388 (12%)	0.0079
Primary diagnosis,				<0.0001
• Infectious	290 (7%)	103 (11%)	187 (6%)	
• Cardiac	1482 (36%)	298 (33%)	1184 (38%)	
• Respiratory	726 (18%)	140 (15%)	586 (19%)	
• Malignant	421 (10%)	168 (18%)	253 (8%)	
• Other / uncertain	1148 (28%)	204 (22%)	944 (30%)	
Duration to death or discharge, days		8 (2–18)	9 (4–17)	
Base excess at admission, mmol/L	0.2 (-4.2–3.9)	-3.4 (-9.2–2.3)	0.8 (-2.9–4.3)	<0.0001
Base excess at 24h, mmol/L	1.1 (-2.2–5.0)	-0.3 (-4.0–3.4)	1.8 (-1.4–5.5)	<0.0001
Base excess minimum in 24h, mmol/L	-1.0 (-5.8–2.8)	-5.7 (-12.1–0.4)	-0.3 (-4.2–3.1)	<0.0001
Base excess minimum in 24-48h, mmol/L	3.2 (0.1–7.2)	1.85 (-1.5–5.8)	3.7 (0.6–7.5)	<0.0001
Base excess slope, mmol/L	-0.3 (-2.0–0)	-0.9 (-3.2–0)	-0.1 (-1.8–0)	<0.0001
Lactate at admission, mmol/L	1.4 (0.9–2.4)	2.3 (1.2–5.8)	1.2 (0.8–2.0)	<0.0001
Lactate at 24h, mmol/L	1.1 (0.8–1.7)	1.5 (0.9–2.5)	1.0 (0.7–1.5)	<0.0001
Lactate maximum in 24h, mmol/L	1.7 (1.1–2.9)	3.0 (1.6–9.0)	1.5 (1.0–2.3)	<0.0001
Lactate maximum in 24-48h, mmol/L	1.4 (0.9–2.1)	2.0 (1.3–3.6)	1.2 (0.8–1.8)	<0.0001
Lactate slope, mmol/L	-0.3 (-1.0–0)	-0.5 (-1.6–0)	-0.3 (-0.9–0)	<0.0001
pH at admission	7.39 (7.33–7.44)	7.35 (7.23–7.43)	7.40 (7.35–7.44)	<0.0001
pH at 24h	7.42 (7.37–7.47)	7.40 (7.33–7.45)	7.43 (7.39–7.47)	<0.0001
pH minimum in 24h	7.37 (7.28–7.41)	7.28 (7.13–7.37)	7.38 (7.32–7.42)	<0.0001
pH minimum in 24-48h	7.45 (7.41–7.49)	7.43 (7.39–7.49)	7.46 (7.42–7.5)	<0.0001
pH slope	0 (-0.05–0)	-0.03 (-0.09–0)	0 (-0.04–0)	<0.0001

Values are n (%) for categorical variables and median (interquartile range) for continuous variables. Differences of groups of patients who died, and patients discharged from hospital alive were tested and p values are reported from χ^2^ test for nominal variables and Mann Whitney U test for continuous variables.

Definition of primary diagnosis groups: Infectious = ICD R57.2 + A + B; Cardiac = ICD R57.0 + I; Respiratory = ICD J; Malignant = ICD C + D; Other / uncertain

ICD-10:

A + B = Certain infectious and parasitic diseases

C + D = Neoplasms and Diseases of the blood and hematopoietic organs and certain disorders involving the immune system

E = Endocrine, nutritional and metabolic diseases

F = Mental and behavioral disorders

G = Diseases of the nervous system

I = diseases of circulatory system

J = Diseases of the respiratory system

K = Diseases of the digestive system

R57.0 = Cardiogenic shock; R57.1 = Hypovolemic shock; R57.2 = Septic shock.

Note: Pneumonia and ARDS classified in category J (disease of respiratory system)

**Table 2:** Characteristics of complete cases (patients with duration of ICU treatment >48 h) compared to patients with ICU treatment <48 h.

	Patients with ICU treatment >48 h	Patients with ICU treatment <48 h	p value
Duration of ICU treatment	5 (3–9) days	21 (14–28) h	
Mortality	404 (24%)	509 (22%)	n.s.
Age	67 (55–78)	68 (55–78)	n.s.
Gender (m, male; f, female)	m 984 (57%)	m 1286 (55%)	n.s.
	f 731 (43%)	f 1066 (45%)	
SAPS II score, points	43 (29–55)	39 (27–52)	0.0001
SAPS II estimated mortality rate, %	31 (10–58)	23 (8–51)	0.0002
Invasive ventilation	1177 (69%)	719 (31%)	<0.0001
Dialysis	364 (21%)	167 (17%)	<0.0001
Primary diagnosis,			n.s.
• Infectious	126 (7%)	164 (7%)	
• Cardiac	596 (35%)	886 (38%)	
• Respiratory	323 (19%)	403 (17%)	
• Malignant	195 (11%)	226 (10%)	
• Other / uncertain	475 (28%)	673 (29%)	
Base excess at admission, mmol/L	0.3 (-4.3–4.0)	0.2 (-4.1–3.8)	n.s.
	AUC for mort. 0.643	AUC for mort. 0.655	
Lactate at admission, mmol/L	1.4 (0.9–2.4)	1.4 (0.9–2.5)	n.s.
	AUROC for mort. 0.679	AUC for mort. 0.713	
pH at admission	7.39 (7.33–7.44)	7.39 (7.32–7.44)	n.s.
	AUROC for mort. 0.617	AUC for mort. 0.639	

Values are n (%) for categorical variables and median (interquartile range) for continuous variables. Differences of groups of patients with duration of ICU treatment >48 h or < 48 h were tested and p values are reported from χ^2^ test for nominal variables and Mann Whitney U test for continuous variables.

For base excess, lactate and pH at admission, AUROC for prediction of mortality is reported additionally (all p <0.0001).

Abbreviations: ICU, intensive care unit; h, hours; n.s., not significant; mort., mortality.

### Univariate analysis: ROC and Cox regression

**Fig 2:**
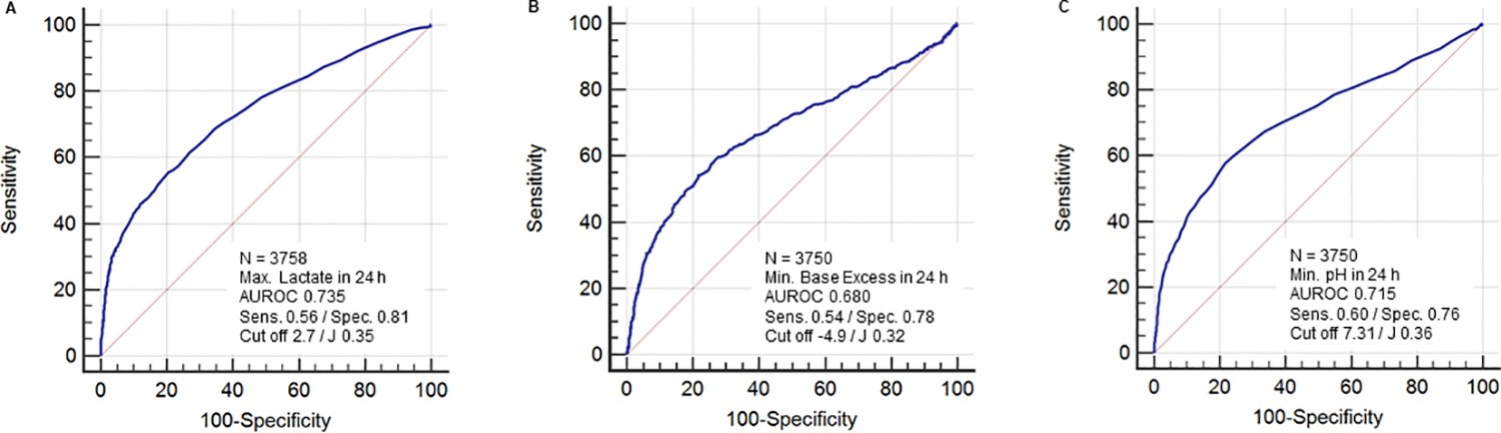
ROC analysis of mortality by maximum lactate (A), minimum base excess (B) and minimum pH (C) in the first 24 h after admission. Abbreviations: AUROC, Area under the receiver operating characteristics curve; max., maximum; min., minimum; sens., sensitivity; spec., specificity; J, Youden-Index.

All evaluated variables (value at admission, value after 24 ± 4 hours, maximum (lactate) or minimum (pH, base excess) value in the first 24 hours and in 24–48 hours after admission and slope of lactate, pH and base excess) were associated significantly with mortality in univariate analysis (Table 3). The variables with the highest area under the receiver operating characteristics curve (AUROC) were maximum lactate in the first 24 hours after admission (AUROC 0.74, sensitivity 0.56 and specificity 0.81 at a cut off value of 2.7 mmol/L) and minimum pH in the first 24 hours after admission (AUROC 0.72, sensitivity 0.60 and specificity 0.76 at a cut off value of 7.31, Fig 2). Lactate, base excess and pH at ICU admission predicted mortality in both subgroups of patients with ICU treatment durations >48 h or <48 h (AUROC in Table 2).

**Table 3:** Univariate correlations with mortality: ROC and Cox regression.

Parameter	N	AUROC	Risk group (Cut off)	HR of risk group	HR per SD(95% CI)
		(95% CI)		(95% CI)	
Age, years	4067	0.575	> 58	1.61	1.35
		(0.560–0.591)		(1.37–1.90)	(1.26–1.46)
SAPS II estimated mortality rate, %	2481	0.529	> 52	1.33	1.10
		(0.509–0.548)		(1.12–1.59)	(1.01–1.19)
Base excess at admission, mmol/L	4030	0.649	< -3.8	2.27	0.69
		(0.635–0.664)		**(**1.99–2.59)	(0.65–0.74)
Base excess at 24h, mmol/L	1922	0.604	< -1.2	1.57	0.79
		(0.583–0.628)		(1.32–1.86)	(0.72–0.86)
Base excess min in 24h, mmol/L	3750	0.680	< -4.9	**2.47**	**0.64**
		(0.665–0.695)		**(**2.15–2.83)	(0.61–0.68)
Base excess min in 24-48h, mmol/L	1905	0.602	< 2.2	1.53	0.76
		(0.580–0.625)		(1.28–1.82)	(0.69–0.84)
Base excess slope, mmol/L	3750	0.589	< -2.4	1.52	0.84
		(0.572–0.604)		(1.31–1.75)	(0.81–0.89)
Lactate at admission, mmol/L	4035	0.698	> 2.1	**2.93**	**1.39**
		(0.683–0.712)		**(**2.57–3.34)	(1.34–1.44)
Lactate at 24h, mmol/L	1925	0.652	> 1.4	2.06	1.26
		(0.632–0.675)		(1.74–2.44)	(1.21–1.32)
Lactate max in 24h, mmol/L	3758	**0.735**	> 2.7	**3.20**	**1.40**
		(0.721–0.749)		(2.79–3.67)	**(**1.35–1.44)
Lactate max in 24-48h, mmol/L	1904	0.702	> 1.7	2.20	1.30
		(0.683–0.724)		(1.84–2.64)	(1.24–1.36)
Lactate slope, mmol/L	3758	0.574	< -1.0	1.62	0.84
		(0.557–0.589)		(1.40–1.86)	(0.80–0.88)
pH at admission	4030	0.630	< 7.31	2.60	0.72
		(0.614–0.645)		(2.28–2.97)	(0.68–0.75)
pH at 24h	1925	0.640	< 7.36	1.89	0.76
		(0.617–0.661)		(1.59–2.26)	(0.71–0.81)
pH min in 24h	3750	**0.715**	< 7.31	**2.94**	**0.64**
		(0.700–0.729)		(2.56–3.38)	(0.61–0.67)
pH min in 24-48h	1902	0.592	< 7.43	1.73	0.77
		(0.569–0.614)		(1.45–2.07)	(0.71–0.83)
pH slope	3751	0.612	< -0.05	1.54	0.95
		(0.597–0.628)		(1.36–1.80)	(0.93–0.98)

Hazard ratios are of risk group defined by cut off from ROC (e.g. risk group with age ≥ 58 years compared to group with age < 58 years) and per standard deviation increase.

Values with p < 0.05 are listed only. There was no significant correlation of gender and mortality. Highest AUROC and highest or lowest hazard ratios are marked in bold.

Abbreviations: AUROC, Area under the receiver operating characteristic curve; HR, hazard ratio; CI, confidence interval; n.s., not significant; min, minimum; max, maximum.

In proportional hazard analyses using the cut-offs from ROC analyses for stratification of risk groups, the highest hazard ratios were found for base excess, lactate and pH at admission and for minimum base excess, minimum pH and maximum lactate in 24 hours after admission (Table 3), with hazard ratio for minimum or maximum values in the first 24 h overall higher than for values at admission. In proportional hazards determined per standard deviation, maximum lactate in 24 h, lactate at admission, minimum base excess in 24 h and minimum pH in 24 h showed highest or lowest hazard ratio per SD (Table 3).

Results of proportional hazard analyses in the subgroups of primary diagnoses overall resembled the results in the total cohort (Table 4): Maximum lactate in 24 h was a strong predictor of mortality in all groups; lactate values were overall strong predictors of mortality, and the interval-related maximum or minimum values of all markers were overall stronger predictors of mortality than values at admission. Additionally, base excess at admission and minimum base excess in 24 h were strong predictors of mortality in the cardiac disease group; and age was a strong predictor of mortality in the respiratory disease group (Table 4).

**Table 4:** Univariate hazard ratios for subgroups of primary diagnosis.

Primary diagnosis	Infectious	Cardiac	Respiratory	Malignant	Uncertain / other
	n = 290	n = 1482	n = 726	n = 421	n = 1148
Age	1.24	1.27	**2.13**	n.s.	**1.61**
	(1.00–1.56)	(1.09–1.49)	**(1.65**–**2.79)**		**(1.40**–**1.85)**
SAPS II score	n.s.	n.s.	n.s.	1.28	n.s.
				(1.05–1.57)	
Base excess at admission	0.76	**0.54**	0.77	0.75	0.75
	(0.62–0.93)	**(0.49**–**0.61)**	(0.66–0.90)	(0.63–0.90)	(0.67–0.85)
Base excess at 24h	n.s.	0.76	0.79	n.s.	0.72
		(0.62–0.93)	(0.69–0.93)		(0.59–0.87)
Base excess min in 24h	0.75	**0.53**	0.73	0.71	0.63
	(0.63–0.90)	**(0.47**–**0.59)**	(0.62–0.86)	(0.60–0.84)	(0.56–0.72)
Base excess min in 24-48h	n.s.	n.s.	0.79	n.s.	0.65
			(0.65–0.95)		(0.52–0.80)
Base excess slope	0.86	0.85	0.83	n.s.	0.83
	(0.77–0.97)	(0.77–0.95)	(0.73–0.96)		(0.77–0.90)
Lactate at admission	1.37	1.46	1.49	1.40	1.33
	(1.23–1.51)	(1.38–1.54)	(1.20–1.79)	(1.21–1.59)	(1.23–1.42)
Lactate at 24h	**1.40**	1.30	1.17	1.18	1.28
	**(1.25**–**1.55)**	(1.16–1.42)	(1.00–1.32)	(1.03–1.37)	(1.19–1.37)
Lactate max in 24h	1.32	**1.53**	**1.52**	**1.56**	**1.46**
	(1.20–1.45)	**(1.44**–**1.62)**	**(1.28**–**1.76)**	**(1.01**–**1.26)**	**(1.36**–**1.57)**
Lactate max in 24-48h	1.34	1.29	1.48	1.14	1.41
	(1.19–1.50)	(1.18–1.39)	(1.13–1.85)	(1.00–1.25)	(1.29–1.52)
Lactate slope	n.s.	0.79	0.78	n.s.	n.s.
		(0.69–0.93)	(0.73–0.84)		
pH at admission	0.72	0.64	0.76	0.85	0.73
	(0.63–0.84)	(0.60–0.69)	(0.66–0.87)	(0.76–0.96)	(0.65–0.82)
pH at 24h	0.70	0.69	0.81	0.78	0.72
	(0.59–0.85)	(0.60–0.80)	(0.72–0.93)	(0.65–0.94)	(0.63–0.85)
pH min in 24h	0.71	0.60	0.66	0.76	0.61
	(0.62–0.82)	(0.55–0.64)	(0.58–0.75)	(0.68–0.86)	(0.54–0.69)
pH min in 24-48h	0.82	0.75	0.75	0.76	0.73
	(0.69–0.98)	(0.64–0.88)	(0.63–0.91)	(0.63–0.93)	(0.61–0.88)
pH slope	0.80	n.s.	**0.54**	0.64	**0.58**
	(0.65–1.00)		**(0.44**–**0.69)**	(0.50–0.84)	**(0.49**–**0.70)**

Values are Hazard ratio per standard deviation increase and 95% confidence interval. Highest or lowest hazard ratios for every group of primary diagnosis are marked in bold. For definition of primary diagnosis groups see Table 1.

n.s. = not significant; for all other tests p-value was < 0.05.

Abbreviations: min, minimum; max, maximum.

### Kaplan Meier curves

**Fig 3:**
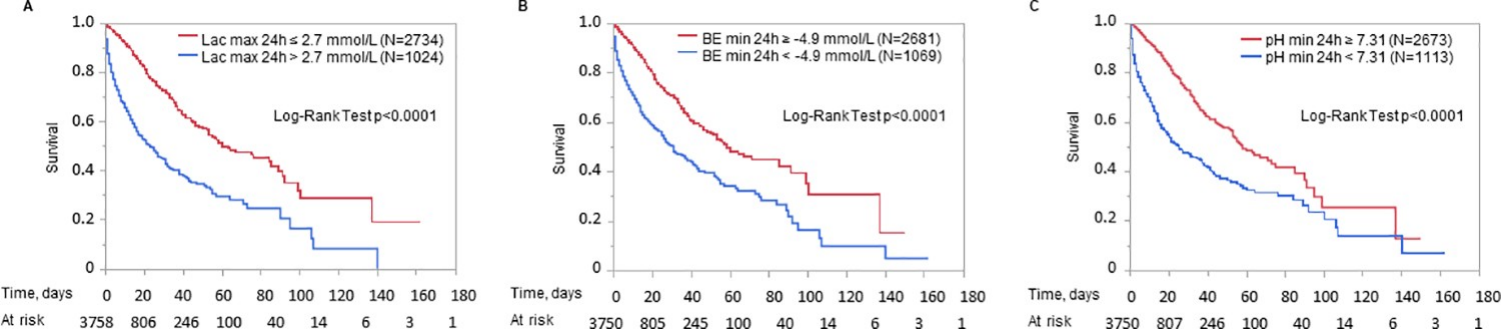
Kaplan Meier curve of mortality by maximum lactate (A), minimum base excess (B) and minimum pH (C) in the first 24 h after admission. Cut off values used for stratification in risk groups were determined by ROC analysis. Abbreviations: Lac, lactate; BE, base excess.

Kaplan Meier curves for groups stratified by cut offs from C-statistics are shown as an example for maximum lactate in 24 h after admission and minimum base excess and minimum pH in 24 h after admission (Fig 3). Kaplan Meier curves for the cut offs maximum lactate in 24 h > 2.7 mmol/l, minimum base excess in 24 h < -4.9 mmol/L and minimum pH in 24 h < 7.31 showed a clear separation particularly in the first 20 days after admission to ICU (Fig 3).

## Discussion

In our cohort of medical ICU patients, all investigated parameters, lactate, pH and base excess, were suitable predictors of ICU mortality. Cut off values at admission to ICU for prediction of mortality were with 2.1 mmol/L for lactate and -3.8 mmol/L for base excess in a range consistent with the values determined in other studies (around 1.5–2.5 mmol/L for lactate and -4 - -6 mmol/L for base excess) [4, 12–14].

However, base excess and pH were not superior to lactate for prediction of mortality in this unselected cohort of medical ICU patients. This is in contrast to patients after heart surgery at admission to ICU, where base excess was superior to lactate for prediction of mortality [7] and trauma patients, where base excess has been found a strong predictor of mortality [9, 14]. Interestingly, in our subgroup of cardiac patients, base excess in addition to lactate was also a strong predictor of mortality. In our analyses of the total cohort of medical ICU patients, lactate was the strongest predictor of ICU mortality, followed by pH: Lactate values showed the highest AUROC in ROC analysis and the highest Hazard ratio per standard deviation increase; pH values had the second highest AUROC in ROC analysis; Kaplan Meier curve stratified by maximum lactate over the first 24 h and minimum pH over the first 24 h showed the clearest separations.

For lactate, prognostic significance of the course or clearance has been evaluated [3, 13, 15–17]. In sepsis patients, lactate at 24 hours was found to be strongest predictor of mortality in serial lactate measurements [17] and early lactate clearance was associated with improved outcome [16]. In other unselected cohorts of ICU patients, mortality was higher in patients developing high lactate levels after more than 24 hours following ICU admission or missing lactate clearance in the first 12 hours [3] and lactate at 24 hours after admission to ICU was strongest for prediction of mortality [18]. There are systematic reviews available, that found that across different ICU cohorts lactate clearance was associated with a better outcome [19, 20]; the significance of the course of lactate was thereby independent of the initial value and it was recommended to monitor the lactate level by measurements every 1 to 2 hours [21]. Lactate-guided therapy with monitoring the course of lactate levels after admission to ICU has been suggested to improve treatment outcome [22]. Our findings are consistent with these reports: In our cohort of medical ICU patients, from all lactate values, maximum lactate during the first 24 hours and during 24 to 48 hours after admission to ICU were strongest predictors for mortality in the total cohort and in primary diagnosis subgroups. Altogether, lactate values both at admission and during 48 hours after admission to the ICU are valuable indicators for prognosis assessment.

This results in the question, whether the course of values in the first hours after initiation of intensive care treatment should also be considered for other markers used for evaluation of mortality risk. In patients with extracorporeal life support after out of hospital cardiac arrest, lactate and base excess both showed best predictive power for values measured 3 h after initiation of extracorporeal life support [23]. In our cohort of medical ICU patients, initial values of pH and base excess were less predictive than values in the first 24 to 48 hours of the ICU stay. The strongest predictors were the maximum or minimum values during the first 24 hours after admission. These were also superior to the slope between value at admission and maximum or minimum value in the first 24 hours. Our study therefore corroborates the prognostic significance of the values of all parameters, lactate, pH and base excess, in the first 24 to 48 hours after admission to intensive care unit compared to the single value at admission.

In out cohort, SAPS II predicted mortality with a low precision (AUROC only 0.529) compared to the original AUROC of 0.86 in the generation cohort from 1993 [24]. The actual mortality rate in our cohort was with 22% lower than the mortality rate estimated by SAPS II (which was 29%). This could be due to improved treatment, highlighting that mortality remains difficult to predict as it is dependent on many influenceable and non-influenceable factors. Estimators used to assess mortality risk should be reviewed repeatedly (as happened by developing SAPS III). Blood gas analysis represents a feasible tool to estimate mortality in clinical routine as it is easy to perform (in contrast to scores using multiple parameters) and can be re-evaluated continuously.

We had a relevant number of patients with short term ICU treatment <48 h. Patients who needed treatment at ICU longer than 48 h had a higher SAPS II estimated mortality rate. However, lactate, base excess and pH at admission to ICU were all suitable to predict mortality irrespective of duration of ICU treatment. Course of lactate, base excess and pH also predicted mortality in all primary diagnosis subgroups. This undermines the value of blood gas analysis for estimation of mortality in all critically ill medical patients.

The study is limited by the retrospective and single-center design. Additionally, the influence of treatment on the investigated biomarkers could only be assessed to a limited extent, whereby the response to therapy is likely to be reflected in the course of the parameters: A better prediction of mortality by biomarkers assessed during the course of ICU treatment, compared with values at admission to ICU, reflects the association of poor response to therapy, and thus poor recovery of organ function and normalization of acid-base balance, with mortality. The study complements previous studies in the field of mortality prediction in critically ill patients by highlighting the analytes pH and base excess in addition to lactate and their course over the first 48 hours after admission to ICU in medical ICU patients.

In conclusion, lactate, pH and base excess proved to be consistently valid parameters for estimating mortality in medical critically ill patients, and monitoring changes in these parameters during the first hours of intensive care treatment can improve the accuracy of mortality estimates.

## Supporting information

S1 File Data set. (XLSX)
